# Genetics of Congenital Anomalies of the Kidney and Urinary Tract: The Current State of Play

**DOI:** 10.3390/ijms18040796

**Published:** 2017-04-11

**Authors:** Valentina P. Capone, William Morello, Francesca Taroni, Giovanni Montini

**Affiliations:** Pediatric Nephrology, Dialysis and Transplant Unit, Department of Clinical Sciences and Community Health, University of Milan, Fondazione IRCCS Ca’ Granda-Ospedale Maggiore Policlinico, 20122 Milano, Italy; valentina.capone@unimi.it (V.P.C.); williammorello82@gmail.com (W.M.); francesca.taroni@policlinico.mi.it (F.T.)

**Keywords:** congenital abnormalities of the kidney and urinary tract (CAKUT), genetics, renal hypodysplasia, next-generation sequencing, copy number variants

## Abstract

Congenital anomalies of the kidney and urinary tract (CAKUT) are the most frequent form of malformation at birth and represent the cause of 40–50% of pediatric and 7% of adult end-stage renal disease worldwide. The pathogenesis of CAKUT is based on the disturbance of normal nephrogenesis, secondary to environmental and genetic causes. Often CAKUT is the first clinical manifestation of a complex systemic disease, so an early molecular diagnosis can help the physician identify other subtle clinical manifestations, significantly affecting the management and prognosis of patients. The number of sporadic CAKUT cases explained by highly penetrant mutations in a single gene may have been overestimated over the years and a genetic diagnosis is missed in most cases, hence the importance of identifying new genetic approaches which can help unraveling the vast majority of unexplained CAKUT cases. The aim of our review is to clarify the current state of play and the future perspectives of the genetic bases of CAKUT.

## 1. Introduction

Congenital anomalies of the kidney and urinary tract (CAKUT) include a wide range of structural malformations resulting from defects in the morphogenesis of the kidney and of the urinary tract [1,2,3,4]. They are the most common form of malformations at birth, affecting 3–7 out of 1000 live births [5] and representing more than 20% of birth defects [6]. CAKUT effect 40–50% of pediatric and 7% of adult end-stage renal disease worldwide [7,8,9]. In a cohort of 312 children affected by renal malformations (excluding cases with isolated ureteric anomalies) who were followed up until the age of 30 years, an overall poor renal survival for CAKUT patients was described, with a lower outcome for patients carrying bilateral renal hypodysplasia, solitary kidney, and posterior urethral valves, compared with other categories [9]. Nevertheless, retrospective data from an Italian cohort of 146 children 0–18 years old with congenital solitary kidney show a decreased estimated glomerular filtration rate in 12% of the population at a median age of 2.2 years, an estimated survival of 82% at 10 years and proteinuria and/or systemic hypertension in less than 5% of the population; these data highlight the importance in renal outcome of an adequate size of the solitary kidney, which, if smaller than expected, can significantly increase the risk of chronic kidney disease (CKD) [10]. Anyhow, children with CAKUT are known to experience a slower progression of CKD compared to those with glomerular disease [11].

CAKUT are usually detected with prenatal sonography but many cases remain undiagnosed until adulthood [4] and represent a large spectrum of disease with different grade of severity and renal outcome. So far the most commonly used classification is based on anatomical characteristics (segment of the urinary tract involved), with ureteropelvic junction obstruction being the most frequent observed phenotype (20%) [4]. All the malformations can be either uni- or bilateral and, when more than one anomaly is present, it may be hard to understand if all the defects occur independently or if one of them is the primum movens of the whole phenotype [12,13,14]. Moreover, CAKUT can appear as an isolated feature or as part of a systemic condition with extra-renal manifestations [15,16,17] making diagnosis and clinical classification even more challenging.

The pathogenesis of CAKUT is based on the disturbance of normal nephrogenesis, secondary to environmental or genetic causes. Several environmental risk factors have been recognized so far, including maternal diabetes [18] and intrauterine exposure to ACE-inhibitors [19]. On the other hand, the observation of kidney and urinary tract malformations in different members of the same family or as a feature of complex genetic syndromes has enlightened the contribution of genetics in CAKUT and is driving research efforts towards its understanding. The increased recurrence risk of CAKUT among relatives has been confirmed in several studies and is estimated at 4–20% [20,21,22,23,24,25,26]. Multigenerational occurrence of disease has suggested multifactorial or dominant inheritance with reduced penetrance in most kindreds [23,24,25,27,28] but families with recessive inheritance have also been reported [26]. Moreover, the observation of CAKUT being relatively common and mostly sporadic suggests that de novo mutations in genes that potentially contribute to CAKUT, could explain the phenotype in most cases [29].

The aim of our review is to clarify the current state of play and the future perspectives of the genetic bases of CAKUT.

## 2. Pathogenesis

The development of the mammalian kidney during embryonic life is a multi-stage process that derives from reciprocally inductive events between two intermediate mesenchymal progenitors: the ureteric bud (UB) and the metanephric mesenchyme (MM). It begins at E10.5–E11 in the mouse and at 35–37 days of gestation in humans with the induction of the ureteric bud from the nephric duct, followed by mesenchymal to epithelial transition (MET) and branching morphogenesis, and terminates with nephron patterning and elongation (which include proximal and distal tubule morphogenesis and glomerulogenesis) [1,12,30,31,32,33,34,35]. These embryologic events require a tight regulation at the DNA level (mediated by transcriptional factors) and the post-transcriptional level [36]. As mentioned above, perturbation in each of these steps, due to exposure to environmental risk factors or to the dysfunction of genes that direct this process, can lead to CAKUT [1]. Moreover, the interdependence between distinct developmental pathways explains why defects in different genes result in similar phenotypes [14,34,37,38,39,40,41,42,43,44], why a mutation in a single gene can have pleiotropic effects and, therefore, why a morphologic classification alone can’t predict the primary genetic defect [4].

## 3. Research Strategies Overview

To better understand the genetic bases of CAKUT, it is useful to review the most commonly used genetic study approaches, which depend on the characteristics of the population study (sample size, number and size of pedigrees) and of the trait of interest (mono or polygenic) [4].

### 3.1. Candidate Gene Approach

It uses large cohorts of sporadic cases or small pedigrees in case-control association studies to find common disease-associated alleles [4].

### 3.2. Linkage Analysis

It uses a single large pedigree that co-segregate genes with large effect or a large number of small pedigrees. It has been successful for mapping rare Mendelian diseases and susceptibility loci of diseases such as Alzheimer disease, insulin dependent diabetes mellitus and breast cancer, but is not adequate in CAKUT, because it is not sufficiently powerful to detect susceptibility loci with a small effect size [29].

### 3.3. Genetic Isolates

These are populations originated from a limited group of founders, with little immigration into the population, which likely inherited few disease-contributing alleles from common ancestors. These mutations can be detected by searching for shared haplotype in affected individuals, through a genetic strategy called linkage disequilibrium mapping [45,46,47]. For instance, Izzi et al. identified a genetic isolate in an Italian Valley in which different glomerular disease occurred at a much higher prevalence compared to the general population and, using linkage disequilibrium mapping, they reconnected most of the patients to a few common founders [48].

### 3.4. Next-Generation Sequencing (NGS) (Whole Genome Sequencing, Whole Exome Sequencing and Targeted Resequencing)

The term NGS (also known as high-throughput sequencing) refers to a group of modern sequencing technologies developed to allow DNA and RNA sequencing in a more time-efficient and cost-effective manner compared to previous methods like Sanger sequencing. NGS can be applied to the whole genome, the whole exome (which is the protein-coding portion of the genome) or to specific genes or loci of interest [49]. For instance, exome sequencing of patient-parent trios can be used to detect de novo mutations, whose paradigm has been recently supported by an increasing rate of de novo mutations in some heterogeneous disorders (intellectual disability, schizophrenia, autisms spectrum) [50,51,52].

### 3.5. Genome-Wide Association Studies

It covers the whole genome to search for causative gene variants [53]. Association studies using a large number of patients and matched controls can be used in sporadic cases of CAKUT with complex genetic aetiology like vesicoureteral reflux (VUR), but, so far, they have not been successful in this field because they need larger sample size to increase the statistical power to reach genome-wide significance [29].

### 3.6. Structural Variants

Copy number variations (CNVs), defined as gain or loss of germ line DNA of a size ranging from 1 kilobase (Kb) to several megabases (Mb) [54] (identified by array-based Comparative Genomic Hybridization (aCGH) and high-density SNP arrays), are a common feature of the human genome [55,56] and have been associated with multiple human phenotypes, including neurodevelopmental diseases [57,58], schizophrenia [59,60], autism [61], epilepsy, cardiac defects [62,63], lung disease, craniofacial malformations, and others [58,61,64,65,66]. In one of the largest studies to date, pathogenic CNVs accounted for 14.2% of cases among 15,767 children with intellectual disability and variable congenital defects [58]. Given that CNVs usually affect the dosage of multiple genes at the same time, the identification of the major genetic drivers underlying such events is usually very challenging [67].

## 4. Genetic Results

Our current knowledge in the genetics of CAKUT is mostly derived from mouse models and syndromic human developmental disease, which are easier to study and have led to the identification of numerous candidate genes of CAKUT in humans [3,29,68]. The hypothesis that CAKUT can be caused by single-gene mutations derives from the observation that some monogenic mouse models can show CAKUT phenotypes and that monogenic human syndromes can comprise CAKUT phenotypes [1,20]. So far, the most important genes identified to be involved in human and murine kidney development are *RET* and *WNT11* (UB specific), *GDNF*, *WT1*, *EYA1* (MM specific) and *PAX2* (expressed in both UB and MM), being the *GDNF/RET* the most frequently studied pathway [34,69,70].

### 4.1. Syndromic Forms

As already mentioned above, syndromic forms were the first group of disease of interest in CAKUT research. Renal-Coloboma Syndrome (renal hypoplasia and coloboma, caused by *PAX2* mutations), Orofaciodigital Syndrome (associated with renal cysts, caused by *OFD1* gene mutations), Branchio-Oto-Renal Syndrome (caused by mutations in *EYA1*, *SIX1* and *SIX5* genes), Renal cysts and Diabetes Syndrome (associated with mutations in *HNF1β* gene), Fraser Syndrome (characterized by eye abnormalities, syndactyly and various CAKUT forms, caused with mutations in *FRAS1*, *FREM2* or *GRIP1* genes), Alagille Syndrome (bile ducts abnormalities, facial features, heart and kidney malformations, for *JAG1* and *NOTCH1* mutations), and Townes–Brocks Syndrome (imperforate anus, hands and ears malformations and kidney abnormalities, caused by *SALL1* mutations) (Table 1) are all known to be caused by point mutations in single genes and show complex phenotypes with various renal and extra-renal involvement [17,71,72,73,74,75]. A better insight in the genetic background of these syndromes allowed to broad the spectrum of genes involved in their pathogenesis, identify genetic pathways of kidney development dysregulation and, in some cases, help to explain the phenotype also in patients with isolated CAKUT without extra-renal syndromic manifestations [44].

### 4.2. Non-Syndromic Forms

Genetic research for non-syndromic CAKUT has been more challenging, but some candidate genes derived from both studies in syndromic disease and from knock-out mouse models have been successfully validated by the identification of mutations in affected individuals (Table 1) [29].

In 1995, the first gene defect described as being causative of CAKUT was a frameshift deletion in *PAX2* in a family with optic nerve coloboma, renal hypoplasia and vesicoureteral reflux [15]. *PAX2* plays a critical role in kidney development and its mutations (more than 55 have been reported so far) can lead to different isolated CAKUT phenotypes [76].

*HNF1β* (hepatocyte nuclear factor 1*β*), a transcription factor involved in the embryogenesis of the pancreas and liver and expressed in the Wolffian duct from a very early stage of kidney development [77], was the second gene to be identified following the discovery of a heterozygous mutation in two siblings with renal cysts and diabetes [16,78] and then reported in individuals with isolated CAKUT [44,79]. Interestingly, several publications show that gene deletion in the 17q12 region (which includes *HNF1β*) results in the clinical combination of autism/schizophrenia and CAKUT [80,81]. Moreover, mutations in *HNF1β* can inhibit *PKHD1* gene expression and may contribute to the formation of renal cysts in humans with MODY5 (maturity-onset diabetes of the young type 5) and congenital cystic abnormalities of the kidney [82].

Many studies confirmed that *PAX2* and *HNF1β* mutations can explain up to 15% cases of CAKUT [44,83,84], making them the most important genes to screen for diagnostic purpose. Mutations of *PAX2* seem to be more frequently associated with renal hypodyplasia, while mutations in *HNF1β* are more frequently associated with cystic kidneys [44,83,84].

The ESCAPE STUDY [44] provided the first broad analysis of renal developmental genes contribution (*HNF1β*, *PAX2*, *EYA1*, *SIX1*, and *SALL1*) in a large cohort of children with renal hypodysplasia. This study showed a high prevalence of *PAX2* and *HNF1β* mutations (15% of the population) and demonstrated that patients with identical mutations or large gene deletions could show variable renal phenotypes. In accordance with similar results from Ulinski et al. [85], the ESCAPE Study indicated that 22% of all children with cystic renal hypodysplasia carried a mutation of *HNF1β*, suggesting to screen *HNF1β* mutations in all the individuals with cystic renal dysplasia.

PAX2, EYA1, and SALL1 all belong to the GDNF-RET signaling pathway, which is required for the normal growth and morphogenesis of the ureteric bud during kidney development [70]. Conversely, BMP4 inhibits GDNF-RET-signaling and is expressed in the mesenchymal cells that surround the Wolffian duct [31]; missense mutations in *BMP4*, demonstrated to affect BMP4 protein function, were identified in five CAKUT patients [86,87]. *RET* mutations, which cause multiple endocrine neoplasia (MEN) syndrome [88] and Hirschsprung disease [89], were also described in bilateral renal hypodysplasia/agenesis cases [90,91] and, in addition, patients with Hirschprung disease are described to carry urinary tract defects [92]. Nevertheless, data regarding the frequency of *RET* as a CAKUT-causing gene are conflicting [90,91].

In a recent study [93] of 7 affected family members with CAKUT, disease-causing mutations were detected in *DSTYK* gene and additional *DSTYK* mutations were identified in 7 out of 311 (2.3%) unrelated patients with CAKUT. *DSTYK* was then proposed as a new CAKUT gene, as it is a dual serine/threonine and tyrosine protein kinase that acts as a positive regulator of ERK phosphorylation downstream of FGF-receptor activation during kidney development and colocalizes with FGF receptors in the ureteric bud and metanephric mesenchyme.

WNT proteins, like WNT9b and WNT4, play a crucial role in mesenchimal-to-epithelial transition (MET) [31,32]. WNT-pathway seems to be partially regulated by SIX2 [94] and mutations in *WNT4* and *SIX2* have been identified in patients with CAKUT [86,95].

Mutations in the genes encoding several components of the renin–angiotensin system such as *AGT* (angiotensinogen), *REN* (renin), *ACE* (angiotensin-converting enzyme), and *AGTR1* (angiotensin II receptor type 1) have been linked to renal tubular dysgenesis [96]. Inactivation of different components of the RAS has been performed in mice with discordant results: *Agtr2* [97] and *Agt* [98] null mice have CAKUT, while *Ace* and *Ren* null mice show normal renal development.

The Uromodulin (*UMOD*) gene encodes the Tamm-Horsfall protein (which is the most abundant physiological urinary protein in humans [99,100] produced by renal tubular cells of the distal loop of Henle) and its mutations have been linked to the pathogenesis of familial juvenile hyperuricemic nephropathy (FJHN), glomerulocystic kidney disease (GCKD) and autosomal dominant medullary cystic kidney disease 2 (MCKD2) [33,101]. Nevertheless, UMOD mutations were not identified in 96 patients with isolated CAKUT, implying that it may represent a very rare etiology for this condition [102].

Hwang et al. [103] investigated the frequency of mutations in 17 known dominant CAKUT-causing genes in a cohort of 749 CAKUT patients and demonstrated that mutations of known CAKUT-causing genes are present in 6% of these families, with *SALL1*, *HNF1β* and *PAX2* being the most prevalent disease causing genes. Their findings also revealed that some variants previously reported as disease-causing could not be accepted because of their lack of segregation within families. *HNF1β* and *PAX2* were seen at lower frequency in this study, which could be explained by the fact that previous studies used CAKUT cohorts preselected for CKD and with severe renal anomalies [44,83,84,85,104]. They did not identify mutations in *SOX17*, *UMOD*, *BMP4*, *SIX1*, and *UPK3A* and, on the other hand, identified *SALL1* mutations in >1% of patients, suggesting that this gene may be implicated in CAKUT etiology more commonly than usually believed [44].

Similarly, Nicolaou et al. [105] recently analyzed, through targeted NGS, 208 candidate genes (selected from studies on familial CAKUT, CAKUT-related multi-organ syndromes, and in vitro and in vivo models) in a phenotypically heterogeneous cohort of 453 CAKUT patients, which comprises the largest set of genes analyzed in a numerous cohort of CAKUT patients. They identified 148 candidate variants in 82 genes in 151 patients but only 5 disease-causing mutations (in *HNF1β*, *PAX2*, *SIX5* and *UMOD* genes) were defined as causal mutations. In this study the contribution of previously implicated genes to CAKUT risk was significantly smaller than expected and as previously described because most variants were excluded or reported as of uncertain significance for the lack of segregation or of pathogenicity evidence. All together their results indicate that the genetic architecture of CAKUT can be more complex than previously suggested.

So far, analysis of multiple genes in parallel using next generation sequencing in patients with CAKUT has demonstrated that <10% patients with isolated CAKUT carry variants in about 20 genes [103,106,107] with insufficient data regarding their causative role [1,4,15,16,17,21,22,74,75,78,86,90,93,95,100,108,109,110,111,112,113,114,115,116,117,118,119]. Approximately 50% of mutations in *PAX2* and microdeletion in *HNF1β* are estimated to occur de novo but evidence for de novo mutations in other genes is currently lacking [76].

All these findings imply that the majority of causes of CAKUT are still unknown, while the list of novel variants requiring functional characterization is extending [29], including variants in *FRAS1*, *FREM2*, *GRIP1*, *ITGA8* and *TRAP1* in which recessive mutations have been previously characterized [120,121] providing evidence that CAKUT might in some cases be an autosomal recessive disease.

### 4.3. Genomic Imbalance

**Figure 1:**
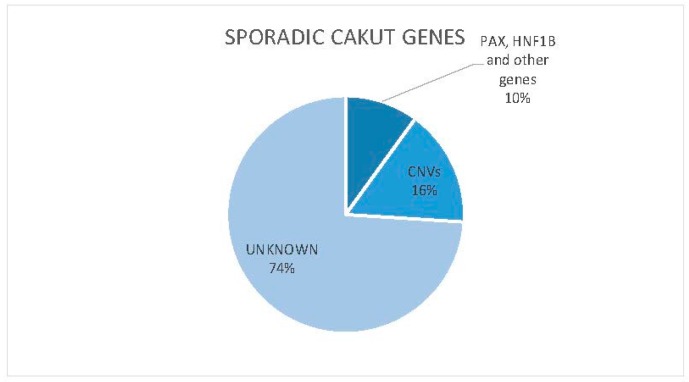
Sporadic *CAKUT* genes.

A new and promising approach in the genetics of CAKUT is copy number variants (CNVs) analysis. Human kidney and urinary tract development is particularly sensitive to gene dosage [122,123,124] and structural genomic defects are increasingly recognized as an important cause of congenital malformations, probably explaining over 16% cases of CAKUT (Figure 1) [125]. For instance, microdeletions of Chromosome 17q12 (which contains *HNF1β* gene), have been described to occur de novo in patients with CAKUT with or without diabetes mellitus [126].

In two different studies the impact of CNVs in CAKUT has been particularly highlighted. In the first one, Sanna-Cherchi et al. [123] compared the frequency of gene-desrupting rare CNVs in two cohorts of patients affected by renal hypodysplasia (522 patients in total) to 4733 controls, demonstrating that rare CNVs account for up to 17% of patients with renal hypodysplasia, then representing a major molecular determinant of kidney malformations. In 2.3% of cases the identified CNV was a rearrangement in chromosomal region 17q12 which contains *HNF1β* gene. The Di George/Velocardiofacial syndrome (also known as 22.q11.2 deletion syndrome) was the next most frequent identified disorder, consistent with the observation that urologic defects occur in about 40% of individuals with this syndrome [127,128], mainly characterized by heart malformations, facial features, hypocalcemia and immune system dysregulation. Interestingly, Sanna-Cherchi identified in 3 cases with isolated renal hypodysplasia a deletion in the distal region of the DiGeorge locus, suggesting that the gene responsible for the urinary-tract defect is likely located in this particular segment. Moreover, 90% of the disorders detected in this study are also known to predispose to developmental delay or neuropsychiatric disease, suggesting shared pathways between renal and neurodevelopmental programs [58].

Westland et al. [129] similarly investigated the role of rare CNVs in 80 patients with solitary functioning kidney from the KIMONO (KIdney of MONofunctional Origin) study population, compared to over 23,000 controls. They identified rare known or new genomic imbalances in 14% of patients and identified five high-priority genetic drivers, proposing *DLG1* and *KIF12* as novel candidate genes for human CAKUT, because of their specific expression in maturing kidney and the development of CAKUT phenotype when mutated in animal models.

## 5. Future Perspectives

Recently, it has been shown that a depletion of miRNAs in different nephrogenic cell lineages in mouse models resembles human CAKUT [36]. MiRNAs are small non-coding RNA molecules of about 22 nucleotides, encoded by >1000 miRNA genes. They function in post-transcriptional regulation of gene expression either by promoting the degradation of mRNA or by inhibiting the translation. Jovanovic [130] recently identified 7 miRs with a potential role in CAKUT, with particular interest for hsa-miR-144. Further functional analysis must be performed to reveal the impact of hsa-miR-144 on CAKUT and to define the precise role of miRs as biomarkers for diagnosis and prognosis of the disease.

## 6. Conclusions

The importance of unraveling the genetics of CAKUT is well-established, since very often CAKUT is only the first manifestation of a complex systemic disease and can manifest in different members of the same family. A precise genetic definition can help the physician identify other subtle clinical features and give the patient and his family an appropriate genetic counseling.

Currently, *HNF1β* and *PAX2* are the primary genes screened for mutations in CAKUT patients. Nevertheless, the number of cases of sporadic CAKUT explained by highly penetrant mutations in a single gene, as previously proposed from earlier studies on patients and animal models, may have been overestimated over the years (Figure 1).

Hence, it is important to identify new and more comprehensive genetic approaches which can help to explain the vast majority of CAKUT cases.

In recent years, along with an increasing interest in gene dosage imbalance as cause of developmental disease, CNVs analysis has become more and more promising in CAKUT genetic research and has allowed to explain another fraction of CAKUT cases (Figure 1).

In the near future, it will be of primary interest to reach a more exhaustive understanding of the genetics of CAKUT. This process will be more feasible as the cost of sequencing declines and as the investigators establish international collaborations to collect larger patient cohorts and share results. The ultimate goal of genetic research in this field will be to reclassify the wide spectrum of CAKUT phenotypes and to stratify renal prognosis (in terms of risk of developing renal failure) accordingly to genotype. Moreover, a better understanding of the biological pathways involved in kidney development dysregulation could lead to the development of innovative targeted therapeutic approaches.

## Figures and Tables

**Table 1:** Genes involved in syndromic and non-syndromic CAKUT.

Gene	Disease
*ACE*	Renal tubular dysgenesis
*AGT*	Renal tubular dysgenesis
*AGTR1*	Renal tubular dysgenesis
*BMP4*	CAKUT
*DSTYK*	CAKUT
*EYA1*	Branchio-Oto-Renal Syndrome and renal hypoplasia
*FRAS1*	Fraser Syndrome
*FREM1*	Bifid nose, renal agenesis, anorectal malformations
*FREM1*	Fraser Syndrome
*GRIP1*	Fraser Syndrome
*HNF1β*	Multicystic dysplastic kidney, renal hypoplasia, renal cysts and diabetes Syndrome
*NOTCH2*	Alagille syndrome, renal anomalies
*PAX2*	Renal coloboma Syndrome and CAKUT
*REN*	Renal tubular dysgenesis
*RET*	Renal agenesis and Hirschsprung disease
*SALL1*	Townes–Brocks Syndrome
*SIX1*	Branchio-Oto-Renal Syndrome
*SIX2*	Renal hypodysplasia
*SIX5*	Branchio-Oto-Renal Syndrome
*SOX17*	CAKUT
*UPK3A*	Renal dysplasia
*WNT4*	Mullerian aplasia and hyperandrogenism
*UMOD*	Familial juvenile hyperuricemic nephropathy (FJHN), glomerulocystic kidney disease (GCKD), Autosomal dominant medullary cystic kidney disease 2 (MCKD2)
